# The Meaning of Autism Friendly in Hospital Settings: A Scoping Review of the Autism Community’s Perspectives

**DOI:** 10.1007/s10803-025-06781-4

**Published:** 2025-03-19

**Authors:** Sarah C G Davenport, Mohammed Alshawsh, Cameron Lee, Alice Garrick, Amanda Brignell, Alexandra Ure, Beth P Johnson

**Affiliations:** 1 Department of Paediatrics, Monash University, Clayton, Australia; 2 School of Psychological Sciences, Monash University, Clayton, Australia

**Keywords:** Autism friendly, Hospital experience, Autism community, Health services accessibility, Healthcare disparities

## Abstract

Hospitals are motivated to create more autism friendly environments to optimise access and experience for the community. However, there is a lack of clarity in what the term autism friendly in hospital settings means. We conducted a scoping review of four online databases and eleven national autism organisations to determine existing definitions for autism friendly within hospital settings. To operationalise the meaning of autism friendly hospital care, we then reviewed barriers and facilitators to hospital care from the perspective of autistic patients. Within the seven studies that considered the meaning of autism friendly, we found that the term autism friendly within a hospital context is undefined. To operationalise the meaning of autism friendly within hospitals, we identified barriers and facilitators in 16 studies that examined the hospital experience of autistic patients. We identified 19 facilitators and 23 barriers across three categories: *people*, *place*, and *time*. Flexibility underpinned the three categories, with flexible *people*, flexible *place*, and flexible *timing* reported as being integral to improving the hospital experience of patients with autism. Our findings provide clear guidance for creating autism friendly hospital care.

Large health disparities exist between individuals with autism spectrum disorder (autism) and the general population (Hwang et al., 2019). These disparities are compounded by numerous barriers that limit an autistic person’s ability to access satisfactory care across a range of healthcare settings (Calleja et al., 2022; Malik-Soni et al., 2022; O’Hagan et al., 2023). Hospitals are a key healthcare setting where accessibility for autistic patients’ needs improvement (Bishop-Fitzpatrick & Kind, 2017). Although individuals with autism require hospital care more frequently (Deavenport-Saman et al., 2016), they are often less satisfied with the care received (Calleja et al., 2022; Malik-Soni et al., 2022; O’Hagan et al., 2023). The barriers to general healthcare are further exacerbated by the inflexibility of hospital systems, with changes to procedures and the physical hospital environment being difficult to implement (Bailey, 2016; Hughes et al., 2022). Additionally, deficiencies in healthcare staff knowledge, resources, and training for caring for autistic patients in hospitals further impact the experience of patients (Garrick et al., 2022; Morris et al., 2019), which is particularly pertinent in tertiary settings reserved for specialised care and emergencies (Garrick et al., 2022). The barriers to care that autistic patients face in hospitals are also experienced in other healthcare settings, such as general practice or dental clinics (Dern & Sappok, 2016), making it an important area to consider first with potentially generalisable results.

Autistic advocacy efforts and a greater awareness of autism have driven the development of initiatives to make settings and services throughout society more autism friendly. These initiatives are designed to support social interaction, sensory and emotion regulation, cognitive needs, and minimise anxiety (Amaze, 2018). The language and frameworks for creating autism friendly services originate in grey literature (i.e. information that has not been commercially published) and online resources, describing factors that increase accessibility of services. However, there is no research-based definition for this term that reflects the diverse needs of the autistic community from their perspective. The colloquial understanding of autism friendly has led to many different descriptions of the term. These either avoid specificity with a focus on encouraging wider inclusion (Autism Spectrum Australia, 2023), or are overly specific leading to a reliance on stereotypes of autism (Matusiak, 2016). The varying understanding of autism friendly limits the systematic and effective implementation of strategies to improve accessibility and create an autism friendly hospital environment. A clearly defined, research-supported definition of autism friendly contextualised to the hospital setting will provide clarity to improve accessibility for autistic patients.

The autistic community is calling for research that includes diverse stakeholder voices and works towards real-world changes to improve the quality of life for autistic people across the lifespan (Roche et al., 2021). The need to clearly define the term autism friendly aligns with this need and contributes to improving hospital care for people with autism. To date, the majority of research within this field has focused on autistic experiences within the broader healthcare environment (Harris et al., 2024; Kouo & Kouo, 2021; Walsh et al., 2021). A need to focus specifically on care in hospitals is highlighted by the over-representation of autistic people presenting for hospital care (Deavenport-Saman et al., 2016; Vohra et al., 2016), the contextual challenges associated with emergency care (Pillai et al., 2023) and the unique sensory challenges associated with hospital settings (Garrick et al., 2022; Samet & Luterman, 2019).

Creating autism friendly hospital environments requires that its definition is applicable for all people with autism. Yet despite the extensive literature on autistic healthcare experiences (Greenwood et al., 2024; Harris et al., 2024; Kouo & Kouo, 2021; Mason et al., 2019, 2021; Walsh et al., 2021), no study has yet defined what the term autism friendly means in hospital settings. Previous healthcare studies often focus on a subpopulation (e.g. children with autism) (Kouo & Kouo, 2021) or do not report characteristics of their sample (Greenwood et al., 2024; Mason et al., 2019), meaning representation of the autism spectrum is unknown. To create an accurate autism friendly definition, the experiences of patients with autism their families, carers, and healthcare professionals must be a focus. Previous research has not focused on the experiences of the autism community (Kouo & Kouo, 2021; Walsh et al., 2021) with a larger emphasis on quantitative outcomes such as procedure completion (Harris et al., 2024). These outcomes fail to describe the impact on wellbeing and the patient’s experience and does not include feedback from the patient and their families on the service provided. Grey literature is important for capturing real-world insights from consumers and community organisations, however it is rarely included (Calleja et al., 2022; Greenwood et al., 2024; Mason et al., 2019). To best capture the experiences of people with autism, previous research has used barriers and facilitators (Calleja et al., 2022; Greenwood et al., 2024; Mason et al., 2019). While barriers and facilitators were explored and some suggestions for improved care were suggested, there was no overarching structure or process for healthcare professionals to use to improve their approach when caring for autistic patients (Calleja et al., 2022; Greenwood et al., 2024; Mason et al., 2019). In this study, we have employed Heyworth et al. (2021)’s framework, which identifies barriers and facilitators that are essential to the quality of life and well-being of autistic people, to create a defined structure for autism friendly hospital care. Barriers and facilitators were grouped under three key areas: (1) *people* (“importance of connected trusting relationships”), (2) *place* (“sensory and social safety”), and (3) *time* (“flexibility to pace and structure… to suit the individual”) (Heyworth et al., 2021). Framing the definition of autism friendly with this model ensures autistic needs are viewed through a strengths-based lens, promoting changes in the hospital system that provide appropriate healthcare for autistic individuals.

The overarching aim of the present study was to review current definitions of autism friendly within a hospital setting. We then further operationalised autism friendly hospital care by presenting barriers and facilitators to care, as identified by the autistic community, and relevant healthcare professionals. Two specific research questions are addressed:

1. What does the term autism friendly mean to autistic individuals, their families, carers, and healthcare professionals in a hospital context?
2. What do autistic individuals, their families, carers, and healthcare professionals view as impeding on and contributing to an autism friendly hospital experience?

We used a scoping review design to synthesise study findings to inform an autism friendly definition and ensure the experiences of the entire autism community are heard, including underrepresented autistic voices, such as those who have co-occurring intellectual disability and/or are non- or minimally-speaking individuals (Happé & Frith, 2020; Leadbitter et al., 2021). We analysed the barriers and facilitators to hospital care as reported by autistic people and their supporters to form an autism friendly definition that is easy to understand and guide improvement in care delivery.

## Methods

We conducted a scoping review to clarify the autism friendly definition and identify evidence to better understand what constitutes autism friendly hospital care. This review followed guidelines by the Joanna Briggs Institute (JBI) (Munn et al., 2018) and adhered to the Preferred Reporting Items for Systematic Reviews and Meta-Analyses extension for Scoping Reviews (PRISMA-ScR) (Peters et al., 2021; Tricco et al., 2018).

### Eligibility Criteria

#### Participants

Studies representing the voices of autistic patients of all ages were included. To ensure representation from across the autism spectrum, we included studies where the patient’s experience was conveyed directly by the patient themselves, or through a parent or carer. Healthcare professionals’ perspectives of their patients’ experiences were also included if they were presented alongside the perspectives of the patient and/or their parent or carer. Studies published from 2013 onwards were considered, reflecting the most recent diagnostic criteria for Autism Spectrum Disorder as defined by the Diagnostic And Statistical Manual Of Mental Disorders, Fifth Edition (APA, 2013).

#### Concept

Included studies focused on autistic patient experience. Experience could include specific barriers and/or facilitators, or a general description. Included studies addressed autism-specific factors that affect the patient when visiting the hospital. Studies that focused on broader healthcare issues, such as service availability and cost of service were excluded.

#### Context

This review centred on the experiences of autistic individuals in general hospitals around the world. Studies that focused on specialist hospital settings, such as dental hospitals, were excluded to avoid including factors specific to that setting (Junnarkar et al., 2023).

#### Type of Evidence Sources

All study types and methods were considered, including peer-reviewed studies with original data (i.e. qualitative, quantitative, or mixed), reviews, commentaries, or book chapters. Grey literature articles from national autism organisations were also considered.

### Search Strategy

To ensure scientific literature and grey literature were represented, we conducted two searches. Search one focused on scientific literature in published and peer-reviewed journals and was performed across four online databases: PubMed, Medline (Ovid), Embase, and PsychInfo. Key terms related to autism, patient experience, and hospital, were used, with search strategy developed by MA in consultation with SD (Table 1).

**Table 1:** Search terms for medline

#	Query
1	exp Autism spectrum disorder/ or Autistic Disorder/
2	Child Development Disorders, Pervasive/
3	(Autis* or Asperger* or pervasive developmental disorder or ASD).ti, ab.
4	1 or 2 or 3
5	Patient Satisfaction/
6	Personal Satisfaction/
7	“Delivery of Health Care”/
8	Mental Health Services/
9	“Outcome and Process Assessment, Health Care”/
10	Health Services Accessibility/
11	Consumer Behavior/
12	Healthcare Disparities/
13	(Patient* or health* or care or personal or consumer* adj1 (satisfaction* or experience* or outcome* or perspective)).ti, ab.
14	(Service* or healthcare adj1 (delivery or utilization)).ti, ab.
15	(Healthcare or care or health or treatment or therap* adj1 (inequalit* or disparit* or inclusive or access* or availability or quality or barriers)).ti, ab.
16	(healthcare experience or lived experience or personal lived experience or disability* accommodations or inclusive or inequality or barriers).ti, ab.
17	(Autis* or sensory or disability* or neurodiver* adj1 (friendly or supportive)).ti, ab.
18	5 or 6 or 7 or 8 or 9 or 10 or 11 or 12 or 13 or 14 or 15 or 16 or 17
19	Health Services/
20	Primary Health Care/
21	“Continuity of Patient Care”/
22	Patient-Centered Care/
23	Hospitals/
24	Emergency Service, Hospital/
25	Inpatients/ or Hospitalization/
26	Outpatients/ or Outpatient Clinics, Hospital/
27	(health service* or healthcare* or care coordination or patient-centered care or patient-centered healthcare or person-centered care or person-centered health care or hospital* or emergency or inpatient* or outpatient).ti, ab.
28	19 or 20 or 21 or 22 or 23 or 24 or 25 or 26 or 27
29	4 and 18 and 28
30	limit 29 to english language

To capture a broader range of perspectives and any existing definitions of the term autism friendly, a second search was conducted focusing on grey literature from national autism organisations, including Autism Canada, Scottish Autism, Autism Europe, Autism New Zealand, Autism Spectrum Australia, Autism Cooperative Research Centre for Living with Autism (Australia), Autism Society (United States), Irish Society for Autism, Autistic Minds (United Kingdom), National Autistic Society (United Kingdom) and Autistic Self Advocacy Network (United States). As there was no advanced search function available on these websites, the search strategy involved separately using the keywords “hospital” and “autism friendly” to explore each organisation’s website, including their resource pages.

### Study Selection and Charting of Data

The screening and extraction process was managed using the Covidence platform (Covidence systematic review software, 2023). All study exclusion decisions at the title and abstract screening stage and full text review stage followed the eligibility criteria which were established a priori. To mitigate bias all studies in the scientific search were screened by SD and either CL or AG. Conflicts at this initial screening stage were included for full text review. A full text review of the potentially relevant studies was completed by SD and one of CL or AG, with above 80% agreement. Conflicts were resolved by consensus between SD and CL. Data extraction template was developed and piloted by SD. Data extraction was completed by SD and CL, with consensus reached through team discussion.

For the grey literature search, both the search and screening were performed by SD. Only a basic search was possible on each website. One website, the Irish Society for Autism, had no search ability so their research articles were screened manually. Studies identified as relevant during the search of national autism organisations were uploaded to Covidence and included in the extraction stage.

### Synthesis of Results

The extracted barriers and facilitators were synthesised using basic qualitative analysis allowing for the frequency of each factor to be determined. Factors were clustered under three categories: *people*, *place*, and *time* as per Heyworth et al. (2021)’s model.

A separate analysis of the supporting evidence was conducted and presented adjacent to the main analysis results. To avoid excluding relevant information, the supporting evidence included studies that had a broader scope than this research (e.g. patients with intellectual disability that included autistic patients, broader healthcare settings that included a hospital context). Only relevant data from these articles were extracted where possible. To avoid duplicated data, the supporting evidence included systematic reviews, meta-analyses, and other review papers. Relevant studies from these reviews that potentially met our inclusion criteria were identified, and the number of studies missed by our search was recorded (Hutchinson et al., 2015).

## Results

### Study Selection

**Fig. 1:**
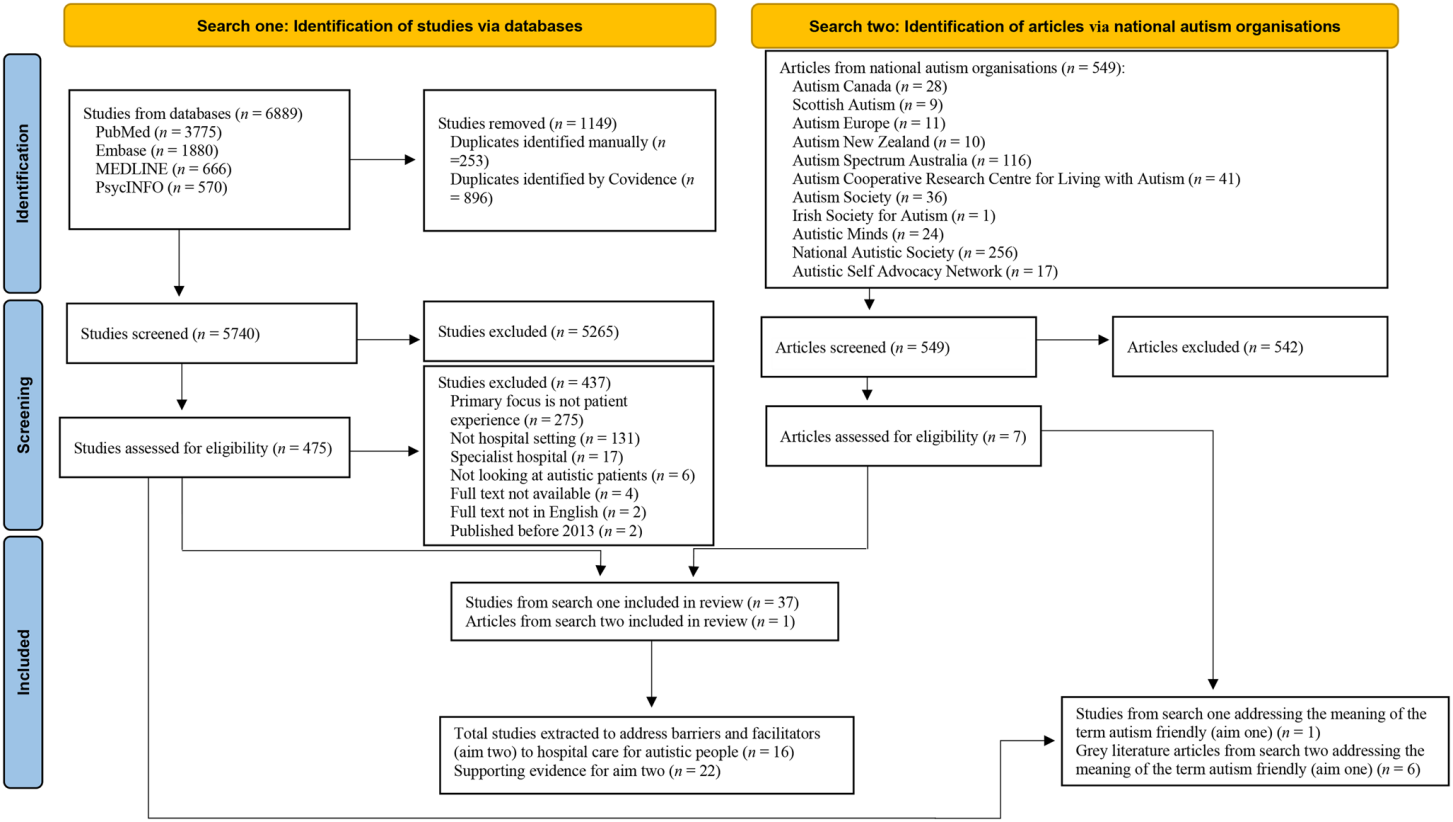
PRISMA diagram

A total of 6,889 studies were identified from scientific databases. At the initial screening stage 5265 studies were excluded as they did not meet the eligibility criteria. After full text review 37 scientific studies met criteria looking at the hospital experience of autistic patients, with 16 studies providing relevant data for the main analysis. The remaining 21 studies were considered relevant but did not have data suitable for the main analysis, these were presented as supporting evidence alongside the main analysis. Of the 37 studies, one study considered the meaning of autism friendly, addressing aim one (Fig. 1).

A total of 549 articles were identified from grey literature. After screening, seven articles met the criteria. Data from these seven articles were analysed separately to distinguish between peer-reviewed data and non-research evidence (Adams et al., 2017). Six articles addressed aim one, looking at the current understanding of autism friendly. One article addressed facilitators to care and was included as supporting evidence as the methodology leading to its outcomes was unclear (Fig. 1).

### Characteristics of Sources of Evidence

The characteristics of the 903 participants included in the 16 studies are summarised in Table 2. Where reported, majority of patients were male (69%) and Caucasian or white (72%). There was limited reporting of autism characteristics and patients’ co-occurring conditions comorbidities, with majority of studies lacking these details. Despite this, a range of autism presentations were represented, including non- and minimally-speaking autistic patients (Table 2). Patients with autism from across the lifespan were represented.

**Table 2:** Characteristics of included studies

Author and year	Country	Data collection method	Participant type (number)		Adult or child patient (age range in years)	Number of male patients (%)	Ethnicity or race of patients	Co-occurring conditions of patient	Characteristics of autism presentation	Hospital department/ reason for hospital visit
Davignon et al. (2014)	United States	Interview		Parents (20 Mo) Healthcare Professionals (20)	Child (3–17)	15 (75%)	Parents; White 80% Black 15% Asian 5%	Anxiety 45% Sleep problems 35% Gastrointestinal problems 25% Genetic syndrome 10% Seizures 10%	Nonverbal 40% Sensory differences 65%	Sedation Unit
Donovan et al. (2023)	Participants from: Australia 4 United Kingdom 3 United States 17	Interview		Patients (24)	Adult (20–49)	0 (0%)	White 83% Mixed 8% Other 8%	Pregnant 100%	NR	Maternity ward
Garrick et al. (2022)	Australia	Survey		Parents (411 Mo 10 Fa^a^)	Child (0–18)	298 (71%)	NR	Anxiety 60.8% ADHD 41.6% Intellectual disability 17.6% ODD 11.9% Eating disorder 5.2% Epilepsy 4.5% Depression 0.9% Cerebral palsy 0.5% Fragile X genetic syndrome 0.2% Sleep disorder 0.2%	Nonverbal 14.7% Single word responses 19.5% Uses group of words 19.0% Uses whole sentences 24.9% Confident and effective verbal skills 21.8% Fluent verbal ability but with social and communication difficulties in ED 11.6% High pain tolerance 55.8%	Emergency Department
Hampton et al. (2022b)	United States 4 United Kingdom 15 Ireland 2	Interview		Patients (21)	Adult (24.9–36.4)	0 (0%)	White 100%	Pregnant 100% Gestational diabetes 19% Polyhydramnios 5% Depression 5% Depression and anxiety 30% OCD and anxiety 10% Other psychiatric conditions 20%	NR	Ultrasound Unit
Hampton et al. (2022a)	United States 4 United Kingdom 19 Ireland 1	Interview		Patients (24)	Adult (21.56–35.76)	0 (0%)	White 100%	Pregnant 100% Gestational diabetes 21% Polyhydramnios 4% Depression 8% Depression and anxiety 29% OCD and anxiety 8% Other psychiatric conditions 21%	NR	Ultrasound Unit
Harwell and Bradley (2019)	United States	Text and Opinion		NR	Child (NR)	NR	NR	NR	NR	Emergency department
Kim et al. (2023)	United States	Interview		Parents (28 Mo; 6 Fa)	Child (2–17)	27 (79%)	White 41% Black < 1% Uknown 47%	NR	NR	Gastrointestinal endoscopic procedure
Kopecky et al. (2013)	United States	Survey		Parents (80)	Adult and Child (2–49)	56 (70%)	White 68% Black 5% Hispanic 4% Asian 6% Other 17%	NR	Minimally verbal (38% used sign language/gestures, 31% used communication tools) Other communication difficulties or preferences (27% exhibited pain through self-injury/aggression)	NR
Menendez (2021)	United States	Interview		Patients (1) Parents (1 Mo; 1 Fa)	Adult (NR)	2 (100%)	NR	NR	NR	Reports on many hospital visits
Muskat et al. (2015)	Canada	Interview		Patients (6) Parents (19 Mo; 3 Fa) Healthcare Professionals (14)	Child (10–16)	17 (85%)	NR	NR	NR	Gastroenterology, neurology, oncology, dentistry, and metabolic genetics
Nicholas et al. (2016b)	Canada	Interview		Patients (4) Parents (24 Mo; 7 Fa) ^b^	Child (3–17)	25 (83%)	Canadian 97% NR 3%	NR	Wide range of autism-related functioning ^c^	Emergency department
Nicholas et al. (2016a)	Canada	Focus groups		Parents (16) ^b^ Healthcare Professionals (44)	Child (3–17)	25 (83%)	Canadian 97% NR 3%	NR	As above	Emergency department
Nicholas et al. (2020)	Canada	Interview		Parents (24 Mo; 7 Fa) Healthcare Professionals (22) ^b^	Child (3–17)	25 (83%)	Canadian 97% NR 3%	NR	As above	Emergency department
Pillai et al. (2023)	Australia	Interview		Caregiver (13 parents; 1 grandparent)	Child (0–18)	8 (57%)	NR	Anxiety disorder 36% ADHD 18% ODD 18% OCD 9% PTSD 9% Specific learning disorder 9% ASD and ID 18%	NR	Emergency department
Snow et al. (2022)	Canada	Interview		Parents (8 Mo) Healthcare Professionals (15)	Child (3–18)	5 (63%)	NR	NR	NR	Ambulatory surgery
Taghizadeh et al. (2019)	Australia	Interview		Caregivers/parents (15) Healthcare Professionals (14)	Child (4–16)	NR	NR	NR	Behavioural challenges Severe 53% Moderate 47% ^d^	Day surgery

Note: NR = not reported in study, Mo = Mothers, Fa = Fathers

^a^ Referred to as male and female parents in Garrick et al. (2022)

^b^ Same participants used

^c^ Interpretation of the Vineland Adaptive Behaviour Scales, Second Edition and the Child Behaviour Checklist scores in Nicholas et al. (2016b)

^d^ Autism severity rated by caregivers based on functioning level and language skills

### Research Question 1: What Does the Term Autism Friendly Mean to Autistic People and Their Families, Carers and Healthcare Professionals in a Hospital Context?

Through our scientific database search (search one; Fig. 1), we found that the term autism friendly was rarely used in the context of hospital settings in scientific studies. Only 10 of the 38 studies referenced the term autism friendly in their study. None of these 10 studies provided a definition or clear explanation of what autism friendly means, however the term was used to indicate the need for autism friendly procedures, environments, and resources. One study by O’Hagan et al. (2023), focused on components that contribute to an autism friendly hospital experience. Although the components outlined environmental/operational modifications and training staff to support autistic patients, the term autism friendly was not defined.

Given the often colloquial nature of the term autism friendly, we also conducted an extensive search of grey literature from the websites of eleven different national autism organisations worldwide (search two; Fig. 1). Every national autism organisation we searched used the term autism friendly or autistic friendly on their website or social media, however none of these sources provided a clear definition for the term. Most notably, an article from Autism Spectrum Australia (2023) featured the perspectives of three autism advocates on their interpretation of autism friendly, two of whom were autistic themselves. To them, autism friendly meant listening to autistic people and responding with kindness, focussing on their strengths, and making small adjustments to be more considerate of autistic needs. While these hold important sentiments, it is difficult to contextualise within hospital settings, and these definitions were represented by a narrow sample of the autism community. In summary, there is currently no definition of what autism friendly means to autistic people, their families, carers, and healthcare professionals within a hospital context.

### Research Question 2: What Do Autistic People and Their Families and Carers, and Healthcare Professionals View as Impeding on and Contributing to an Autism Friendly Hospital Experience?

In total, 19 facilitators to hospital care for autistic patients were identified through 15 studies and 23 barriers to hospital care for autistic individuals were identified throughout 16 studies (Table 3). The barriers and facilitators were synthesised into 9 key factors, forming the three categories (*people*, *place*, and *time;* Heyworth et al., 2021). Barriers and facilitators were consistent throughout studies, with most studies identifying barriers in all three categories.

**Table 3:** Barriers and facilitators identified in studies

		Davignon et al. (2014)	Donovan et al. (2023)	Garrick et al. (2022)	Hampton et al. (2022a)	Hampton et al. (2022b)	Harwell and Bradley (2019)	Kim et al. (2023)	Kopecky et al. (2013)	Menendez (2021)	Muskat et al. (2015)	Nicholas et al. (2016b)	Nicholas et al. (2016a)	Nicholas et al. (2020)	Pillai et al. (2023)	Snow et al. (2022)	Taghizadeh et al. (2019)
**People**	**Healthcare Professionals**																
	Autism-Specific Training and Knowledge	⍟		◯	⍟	◯	⍟	★		⍟	⍟	⍟	⍟	⍟		⍟	◯
	Stigma and Judgement			◯	◯	◯						◯		◯	◯	◯	
	Kindness and Compassion in Care		★		★			★				★		★		★	
	**Communication**																
	Healthcare Professional and Patient	⍟	★	⍟	⍟	⍟	★	◯	★	⍟	◯	⍟		◯		★	★
	Healthcare Professional and Parent			◯			★			⍟		◯	★	◯			◯
	Staff Members	⍟		⍟	◯		★	★		◯	⍟	★	★	⍟		★	★
	**System And Support**																
	Lack of Systems to Support Patient’s Needs	◯		◯						◯	◯			◯	◯		
	Inflexibility from Staff and System									◯	◯		◯				
	Preparatory Materials	◯				★	★	⍟	★	⍟	★	★				⍟	★
	Consistent Care	★			★	★											
	Understanding/Inquiring about Unique Needs	★	★	★		★	★	★		★	★	★	★			★	★
**Place**	**Awareness of and Adjusting for Needs**																
	Unaware of Need for Sensory Modifications	◯		◯	◯										◯		
	Flexible Environment	★					★									★	
	Easy to Understand and Navigate Environment								★	★		★	★	★			
	**Specific Sensory Sensitivities**																
	Noise/Auditory Stimulation		⍟	◯	⍟	◯		★	⍟	◯	⍟	◯	★	★		⍟	◯
	Sight/Lights		⍟	⍟	★	⍟		★	◯	★	◯	◯			◯	⍟	◯
	Touch		⍟	⍟	◯				◯		◯	◯	★		◯	◯	
	Smell		◯	⍟					◯	◯	◯					◯	
	Taste			◯					◯		◯					◯	
	Sensory Discomfort			◯	◯					◯				◯			
	**Distraction And Regulation Materials**																
	Hospital Provided Materials	★	★		★	★	★	★	★	★	★	⍟	◯		◯		★
**Time**	**Pre-Visit Factors**																
	Past Traumatic Experience			◯						◯		◯					
	Preparation Materials	★				★		★			★					★	★
	**Pre-Assessment Factors**																
	Wait Times	⍟		◯			⍟	⍟		◯	◯	⍟	◯		◯		⍟
	Uncertainty				◯	◯				◯			◯				
	Transitioning Throughout Hospital										◯						
	**During Assessment Factors**																
	Behavioural Disruption			◯					◯			◯		◯	◯	◯	◯
	Time Constraints in Appointment	◯										◯					
	Difficulty Communicating Experience			◯	◯	◯			◯		◯	◯				◯	
	Flexibility in Procedure	★	★		★	★	★	★	★	★	★	★					★

Note: ◯ = study identified factor as a barrier, ★ = study identified factor as a facilitator, ⍟ = study identified factor as both a barrier and facilitator

#### Category 1: People

The *people* category describes factors related to the staff and systems within hospitals and were consistently described as a key factor influencing care and experiences. *People* were identified as a facilitator in 15 studies and as a barrier in 14 studies (Table 3). The importance of people in creating autism friendly hospitals was also reflected in the supporting evidence with 19 studies identifying people as a facilitator and 19 studies identifying it as a barrier (Table S1).

Healthcare professionals were identified in 11 studies as facilitating hospital care when they had autism-specific training and knowledge and provided care with kindness and compassion (Table 3). Conversely, healthcare professionals with a lack of training and knowledge about autism and those who hold prejudicial beliefs about autism were the most commonly identified barrier to care as identified in 13 studies (Table 3). This was also reflected in the supporting evidence where 17 studies identified healthcare professionals as preventing autism friendly hospital care (Table S1).

Communication was the most commonly identified facilitator, seen in all 15 studies (Table 3). Effective communication included healthcare professionals being flexible in their approach with a patient, seeking and listening to parent expertise, and sharing understanding and knowledge between staff. The importance of communication was reflected in the supporting evidence where 17 studies identified this factor contributing towards autism friendly care (Table S1). Conversely, poor communication between the healthcare professionals and the patients, parents, or other staff members created frustration for the patient and their supports.

A supportive hospital system facilitated care in 14 studies by providing consistent care (e.g. developing a relationship with one healthcare professional), hospital staff that understood and asked about the particular needs of the patient, and preparatory materials pre-visit (Table 3). In contrast, a rigid hospital system with limited preparatory materials to support the patient’s needs and challenges was identified as barriers. The importance of a supportive hospital system was mirrored in the supporting evidence with 17 studies identifying it as a facilitator and 18 as a barrier (Table S1).

#### Category 2: Place

The *place* category is used to describe the environmental factors affecting autistic patients and the hospitals awareness and ability to support these needs. *Place* was identified as a facilitator in 13 studies and as a barrier in 14 studies (Table 3).

Eleven studies found that when sensory sensitivities are accommodated for, the hospital experience for the patient with autism improved (Table 3). Alternatively, 12 studies reported that when sensory accommodations were not made, it was a barrier to care (Table 3). Garrick et al. (2022) found that 90% of participants identified the sensory environment as an important contributor to stress levels during their experience of hospital visit. The included studies identified each of the five senses - sight, smell, touch, hearing, and taste - as influential to the sensory experiences as well as general sensory input. Eleven studies found that patients’ hospital experiences improved when their sensory sensitivities were able to be accommodated (Table 3).

An environment that was simple and flexible, so that it was easy to navigate and adjust with reasonable accommodations for auditory, visual, tactile and olfactory stimulation was reported to facilitate care. Twelve studies identified barriers to care associated with rigid environments that were unable to accommodate sensory preferences (Table 3). When sensory sensitivities were not accommodated, patients were left feeling overstimulated and in a state of ‘sensory overload’ (Hampton et al., 2022b).

Providing distraction and regulation resources can help autistic individuals feel comfortable and calm throughout their visit. However, these materials were reported to be insufficient, which meant patients remained in a state of overwhelm due to the hospital environment.

#### Category 3: Time

The *time* category refers to balancing the structured timing at the hospital with patients being able to move at their own pace. *Time* was identified as a facilitator in 12 studies and a barrier in 15 studies (Table 3).

Using preparation materials such as social stories (Muskat et al., 2015; Snow et al., 2022) help patients with autism feel informed and prepared, reducing uncertainty and the time needed to adjust to the hospital environment. Trauma associated with previous hospital experiences was identified to negatively affect future hospital experiences. This trauma reportedly impacted the length of time for future hospital visits, either requiring a longer stay to ensure comfort or a shorter one to minimise exposure to triggers.

Wait times were also identified as an important contributor to hospital experience. Shorter wait times meant less exposure to the over-stimulating waiting areas and other autism-specific triggers, resulting in lower levels of anxiety associated with hospital procedures. Twelve studies identified pre-assessment factors, including wait times, lack of preparation, and an unpredictable environment, as barriers to care that can exacerbate uncertainty and anxiety (Table 3).

A flexible approach to hospital visits and procedures, such as allowing longer appointment times, was considered important in 11 studies, enabling autistic patients to feel more comfortable in their environment and with the procedure (Table 3). Behavioural disruption and the time constraints associated with clinicians rushing through health procedures were identified as barriers during appointments. Barriers can become intertwined and exacerbated when considered together. For example, some autistic patients report difficulties communicating their experience, which, when combined with a high pain threshold and not displaying typical pain indicators, may mean that their needs are overlooked. In scenarios where there is limited time, non-typical displays of pain may not be sufficiently considered, increasing the likelihood that the patient and their family feel frustrated and underserved.

## Discussion

Although there is an informal, community understanding of the term autism friendly, there is no evidence-informed definition that can be contextualised in a hospital setting. To establish a more comprehensive definition for autism friendly that offers valuable insights for improving hospital practice, we identified barriers and facilitators to hospital care as reported by autistic patients, their families and carers, and healthcare professionals. Three categories, *people*, *place*, and *time* (Heyworth et al., 2021), contained 19 facilitators and 23 barriers to hospital care for autistic patients. Reported barriers demonstrated a consistent need for healthcare professionals and hospital systems to better understand autism and autistic needs. Reported facilitators consistently outlined a need for healthcare professionals and the hospital system to be flexible and provide accommodations for autistic needs. A definition of autism friendly hospital care must emphasise this by promoting education that creates autism awareness and accommodating for individual needs.

**Fig. 2:**
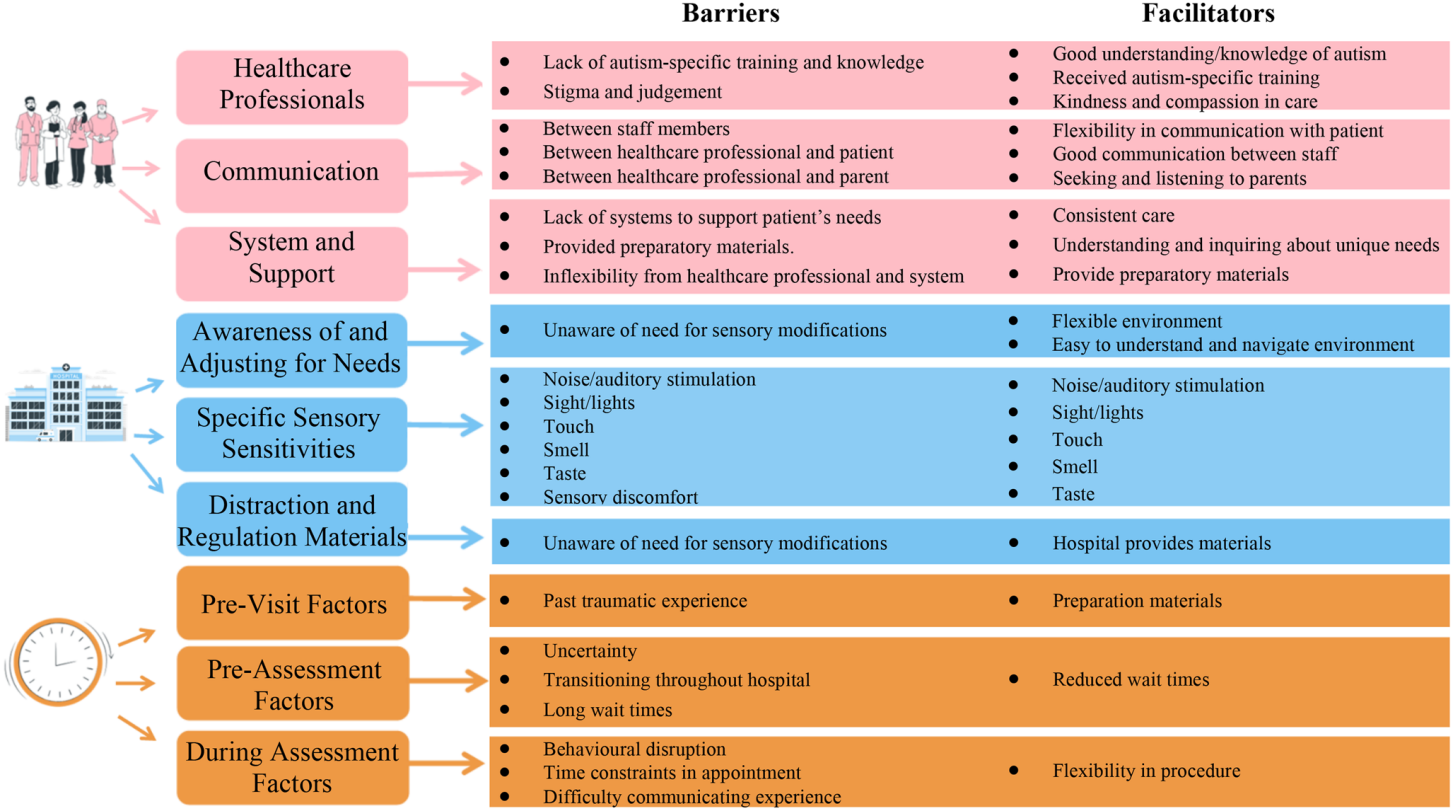
People, place, and time representing factors essential to autism friendly

Autistic patients experienced similar barriers and facilitators evident across the range of age, comorbid conditions, and presenting characteristics of the patients with autism included in our study. The homogeneity of factors suggests they are autism-specific and do not represent wider healthcare access issues (e.g. location, cost). Many factors were identified by multiple participants within each study, reinforcing a similar experience despite differences of presentations. Notably, barriers and facilitators frequently overlap (Fig. 2), with some factors identified as both a barrier and facilitators (e.g. poor communication opposed to flexible communication). Barriers were typically presented as the regular experience, highlighting that current hospital practices are not meeting the needs of autistic patients (Hughes et al., 2022). Comparatively, facilitators were identified as a rare experience or as a suggestion to overcome a barrier. Developing an autism friendly definition informed by these factors will ensure a focus on the experiences for people across the autism spectrum and will clearly indicate where and how improvements should be implemented.

A flexible approach is key to autism friendly hospital care and is seen across the three categories: *people*, *place* and *time*. Given the heterogeneity of autism, a personalised and tailored approach to care is needed to improve the hospital experience and optimise outcomes (Lord et al., 2022). To best understand and accommodate their unique needs, each patient or their caregiver should be asked about their preferences and support needs to create an optimal and autism friendly hospital experience (Wood et al., 2019). Flexibility is then required to implement the necessary adjustments to accommodate for the patient.

Healthcare professionals can incorporate a flexible approach and adjust their communication style to suit their patient. Potential alternative communication options could be explored including writing things down, using picture exchange communication systems (PECS), an electronic device, and/or sign language (Davignon et al., 2014; Donovan et al., 2023; Harwell & Bradley, 2019; Kopecky et al., 2013). Existing initiatives that target communication, such as Scope Australia’s communication access initiative (Scope Australia, n.d.), demonstrate how knowledge, skills, and confidence in communication can increase accessibility for many patients.

A flexible *place* is being able to adjust the environment to suit the needs of the patient. This includes sensory accommodations, such as dimmable lights and noise-reducing doors (Harwell & Bradley, 2019; Nicholas et al., 2020), and ensuring the environment is easy to navigate (Nicholas et al., 2016a, b). Autism friendly spaces are consistent with universal design principles which create adaptable environments that meet individual needs (Milton et al., 2017). Universal design has a focus on incorporating the needs of all users from design inception (Victorian Health Building Authority, 2022) to optimise suitability (Cristiana Palazzo et al., 2024; Imrie, 2014). Including the established universal design strategy within the definition of autism friendly ensures all can benefit regardless of diagnosis and/ or disclosure.

Flexible *timing* involves reducing wait times for autistic patients to lessen uncertainty and minimise exposure to overstimulating waiting areas (Garrick et al., 2022; Harwell & Bradley, 2019; Menendez, 2021; Nicholas et al., 2016b), and allowing for longer appointments (Davignon et al., 2014; Garrick et al., 2022; Hampton et al., 2022a). Flexibility is not effective without knowing each patient’s unique needs and understanding the impact of not meeting these needs. Hence, healthcare professionals must have good knowledge, understanding, and awareness of autism to provide autism friendly care (Nicholas et al., 2016a, b, 2020). To be autism friendly within the hospital setting, healthcare professionals must be educated on autistic patients’ needs so flexible *people*, *place* and *timing* can be implemented.

*People*, *place*, and *time* represent target areas for hospitals to work towards, with barriers and facilitators illustrating the opposing effects of each factor Fig. 2). Previous models tended to be either too broad, leading to reliance on stereotypes, or too specific missing the nuances of the full autism spectrum (Autism Spectrum Australia, 2023; Matusiak, 2016). Having three main target areas assists hospitals with implementation and keeps a wide view to ensure all autistic people and their needs are represented. The inclusion of individual factors, promoting flexibility and education, emphasises the importance of an individualised, patient centred approach and a continuous commitment to agility and improvement. From our research, autism friendly hospital care means that hospitals are inclusive, understanding, flexible, and responsive to the unique needs of each autistic patient.

### Limitations and Future Directions

This study set out to represent the perspectives of individuals from across the autism spectrum. However, the included studies only represented direct voices from 33 patients with autism, with the majority of autistic voices represented by parents/caregivers (870 participants, 96%). The lack of autistic patients’ direct involvement in research was pre-empted, as a known wider issue (Happé & Frith, 2020; Leadbitter et al., 2021). We included parents and families as a voice for autistic people to ensure representation from the full autism spectrum, including those with communication and intellectual challenges. However, the majority of studies included in our review did not report individual demographics, making it difficult to determine the breadth of the autism spectrum represented. Where demographics and autism presentation were reported, patients were majority white and male but represented varying levels of verbal ability, sensory sensitivity, and other autism characteristics.

Our scoping review provides an important first step in defining autism friendly hospital care. Drawing upon perspectives from the autistic and autism community, we have summarised the current understanding and identified a wide range of factors that impact hospital care for autistic people. Following scoping review guidelines, the next step is for these results to be verified in further research (Tricco et al., 2018). Future work should ensure diverse representation across the full autism spectrum, including a range of autism presentations, ages, cultures, locations, and other demographic factors. Additionally, research should focus on determining best practices for autism friendly hospital care, including operationalising these approaches and measuring their impact on patient care, experience and outcomes.

## Conclusion

Our scoping review demonstrated that the term autism friendly is not yet clearly defined within a hospital setting. We therefore set out to operationalise the meaning of autism friendly hospital care, by identifying a wide range of factors reported to impact hospital experiences and care for autistic people. Several factors were consistently reported across studies, suggesting they may be broadly applicable to autistic patients across the spectrum. Our findings suggest that autism friendly hospital care means consideration of *people*, *place* and *timing*, with particular emphasis on adopting a flexible approach to care, enhancing knowledge and understanding of autism, and tailoring care to a patient’s unique needs.

## References

[CR1] Adams, R. J., Smart, P., & Huff, A. S. (2017). Shades of grey: Guidelines for working with the grey literature in systematic reviews for management and organizational studies. *International Journal of Management Reviews*, *19*(4), 432–454. 10.1111/ijmr.12102

[CR2] Amaze (2018). *Amaze position statement: Accessible environments for autistic people*. *Amaze*. https://www.amaze.org.au/wp-content/uploads/2019/06/Amaze-Accessible-environments-March-2018.pdf

[CR3] American Psychiatric Association (2013). *Diagnostic and Statistical Manual of Mental Disorders* (5 ed.). 10.1176/appi.books.9780890425596

[CR4] Autism Spectrum Australia (2023). *What can an individual do to make the world more autism-friendly?* Autism Spectrum Australia. https://www.autismspectrum.org.au/blog/what-can-an-individual-do-to-make-the-world-more-autism-friendly

[CR5] Bailey, L. (2016). Making Macclesfield Hospital accessible to autistic people. https://www.autism.org.uk/advice-and-guidance/professional-practice/accessible-hospital

[CR6] Bishop-Fitzpatrick, L., & Kind, A. J. H. (2017). A scoping review of health disparities in autism spectrum disorder. *Journal of Autism and Developmental Disorders*, *47*(11), 3380–3391. 10.1007/s10803-017-3251-9 28756549 10.1007/s10803-017-3251-9 PMC5693721

[CR7] Boshoff, K., Bowen-Salter, H., Gibbs, D., Phillips, R. L., Porter, L., & Wiles, L. (2021). A meta-synthesis of how parents of children with autism describe their experience of accessing and using routine healthcare services for their children. *Health and Social Care in the Community*, *29*(6), 1668–1682. 10.1111/hsc.13369 34155717 10.1111/hsc.13369

[CR8] Calleja, S., Islam, F. M. A., Kingsley, J., McDonald, R., & Wane, D. (2020). Healthcare access for autistic adults A systematic review. *Medicine (United States)*, *99*(29), E20899. 10.1097/MD.0000000000020899 10.1097/MD.0000000000020899 PMC7373620 32702830

[CR9] Calleja, S., Kingsley, J., Amirul Islam, F. M., & McDonald, R. (2022). Barriers to accessing healthcare: Perspectives from autistic adults and carers. *Qualitative Health Research*, *32*(2), 267–278. 10.1177/10497323211050362 34906008 10.1177/10497323211050362

[CR10] Camm-Crosbie, L., Bradley, L., Shaw, R., Baron-Cohen, S., & Cassidy, S. A. O. (2019). People like me don’t get support’: Autistic adults’ experiences of support and treatment for mental health difficulties, self-injury and suicidality. *Autism: the International Journal of Research and Practice*, *6*(23), 1461–7005. 10.1177/1362361318816053 10.1177/1362361318816053 PMC6625034 30497279

[CR11] Cristiana Palazzo, A., Bertelli, M., Gibertoni, C., Molteni, P., & Gorla, F. (2024). Redefining Hospital Accessibility: A Service Design Framework for Inclusive Healthcare. *IOP Conference Series: Earth and Environmental Science*, *1402*(1), 012066. 10.1088/1755-1315/1402/1/012066

[CR12] Davignon, M. N., Friedlaender, E., Cronholm, P. F., Paciotti, B., & Levy, S. E. (2014). Parent and provider perspectives on procedural care for children with autism spectrum disorders. *Journal of Developmental and Behavioral Pediatrics: JDBP*, *35*(3), 207–215. 10.1097/DBP.0000000000000036 24662617 10.1097/DBP.0000000000000036

[CR13] Deavenport-Saman, A., Lu, Y., Smith, K., & Yin, L. (2016). Do children with autism overutilize the emergency department?? Examining visit urgency and subsequent hospital admissions. *Maternal and Child Health Journal*, *20*(2), 306–314. 10.1007/s10995-015-1830-y 26520161 10.1007/s10995-015-1830-y

[CR14] Dern, S., & Sappok, T. (2016). Barriers to healthcare for people on the autism spectrum. *Advances in Autism*, *2*(1), 2–11. 10.1108/AIA-10-2015-0020

[CR15] Donovan, J., Chiatti, B. D., McKeever, A., Bloch, J. R., Gonzales, M. S., & Birati, Y. (2023). Yes, I can bond. Reflections of autistic women’s mothering experiences in the early postpartum period. *Womens Health (Lond)*, *19*, 17455057231175312. 10.1177/17455057231175312 37209090 10.1177/17455057231175312 PMC10201178

[CR16] Garrick, A., Lee, M. L., Scarffe, C., Attwood, T., Furley, K., Bellgrove, M. A., & Johnson, B. P. (2022). An Australian Cross-Sectional survey of parents’ experiences of emergency department visits among children with autism spectrum disorder. *Journal of Autism and Developmental Disorders*, *52*(5), 2046–2060. 10.1007/s10803-021-05091-9 34061310 10.1007/s10803-021-05091-9

[CR17] Greenwood, E., Cooklin, A., Barbaro, J., & Miller, C. (2024). Autistic patients’ experiences of the hospital setting: A scoping review. *Journal of Advanced Nursing*, *80*(3), 908–923. 10.1111/jan.15880 37743597 10.1111/jan.15880

[CR18] Gupta, N., Brown, C., Deneke, J., Maha, J., & Kong, M. (2019). Utilization of a novel pathway in a tertiary pediatric hospital to Meet the sensory needs of acutely ill pediatric patients. *Frontiers in Pediatrics*, *7*(no pagination). 10.3389/fped.2019.00367 10.3389/fped.2019.00367 PMC6742947 31555627

[CR19] Hampton, S., Man, J., Allison, C., Aydin, E., Baron-Cohen, S., & Holt, R. (2022a). A qualitative exploration of autistic mothers’ experiences I: Pregnancy experiences. *Autism*, 13623613221132435. 10.1177/13623613221132435 10.1177/13623613221132435 PMC10291382 36325726

[CR20] Hampton, S., Man, J., Allison, C., Aydin, E., Baron-Cohen, S., & Holt, R. (2022b). A qualitative exploration of autistic mothers’ experiences II: Childbirth and postnatal experiences. *Autism*, *26*(5), 1165–1175. 10.1177/13623613211043701 34482747 10.1177/13623613211043701 PMC9340136

[CR21] Happé, F., & Frith, U. (2020). Annual research review: Looking back to look forward– changes in the concept of autism and implications for future research. *The Journal of Child Psychology and Psychiatry*, *61*(3), 218–232. 10.1111/jcpp.13176 31994188 10.1111/jcpp.13176

[CR22] Harris, H. K., Weissman, L., Friedlaender, E. Y., Neumeyer, A. M., Friedman, A. J., Spence, S. J., Rotman, C., Krauss, S., Broder-Fingert, S., & Weitzman, C. (2024). Optimizing care for autistic patients in health care settings: A scoping review and call to action. *Academic Pediatric*, *24*(3), 394–407. 10.1016/j.acap.2023.11.006 10.1016/j.acap.2023.11.006 PMC12283146 37951351

[CR23] Harwell, C., & Bradley, E. (2019). Caring for children with autism in the emergency department. *Pediatric Annals*, *48*(8), e333–e336. 10.3928/19382359-20190725-01 31426102 10.3928/19382359-20190725-01

[CR24] Haydon, C., Doherty, M., & Davidson, I. A. (2021). Autism: Making reasonable adjustments in healthcare. *British Journal of Hospital Medicine (London, England: 2005)*, *82*(12), 1–11. 10.12968/hmed.2021.0314 34983215 10.12968/hmed.2021.0314

[CR25] Hemsley, B., & Balandin, S. (2014). A metasynthesis of patient-provider communication in hospital for patients with severe communication disabilities: Informing new translational research. *Augment Altern Commun*, *30*(4), 329–343. 10.3109/07434618.2014.955614 25229213 10.3109/07434618.2014.955614 PMC4266100

[CR26] Heyworth, M., Brett, S., Houting, J. D., Magiati, I., Steward, R., Urbanowicz, A., Stears, M., & Pellicano, E. (2021). It just fits my needs better: Autistic students and parents’ experiences of learning from home during the early phase of the COVID-19 pandemic. *Autism & Developmental Language Impairments*, *6*, 239694152110576. 10.1177/23969415211057681 10.1177/23969415211057681 PMC9620701 36381526

[CR27] Hughes, H., Brown, C., Molan, J., Smith, M., Steele-John, J., Griff, S., Bilyk, C., Thomson, J., Raine, A., Youhorn, P., Campbell, K., Osborne, A., Perks, M., Thomson, R., Monaghan, J., Oberman, S., & Grant, C. (2022). *Services, support and life outcomes for autistic Australians*. Parliament House. https://www.aph.gov.au/Parliamentary_Business/Committees/Senate/Autism/autism/Report/section?id=committees%2freportsen%2f024412%2f77833 Commonwealth of Australia Retrieved from.

[CR28] Hutchinson, J., Prady, S., Smith, M., White, P., & Graham, H. (2015). A scoping review of observational studies examining relationships between environmental behaviors and health behaviors. *International Journal of Environmental Research and Public Health*, *12*(5), 4833–4858. 10.3390/ijerph120504833 25950651 10.3390/ijerph120504833 PMC4454941

[CR29] Hwang, Y. I. J., Srasuebkul, P., Foley, K. R., Arnold, S., & Trollor, J. N. (2019). Mortality and cause of death of Australians on the autism spectrum. *Autism Research*, *12*(5), 806–815. 10.1002/aur.2086 30802364 10.1002/aur.2086

[CR30] Imrie, R. (2014). Designing inclusive environments and the significance of universal design. In J. Swain, S. French, C. Barnes, & C. Thomas (Eds.), *Disabling barriers, enabling environments* (pp. 287–294). Sage.

[CR31] Junnarkar, V. S., Tong, H. J., Hanna, K. M. B., Aishworiya, R., & Duggal, M. (2023). Qualitative study on barriers and coping strategies for dental care in autistic children: Parents’ perspective. *International Journal of Paediatric Dentistry*, *33*(2), 203–215. 10.1111/ipd.13035 36271894 10.1111/ipd.13035

[CR32] Kim, T., Martinez, K., Cruz, B. L., Huang, J. S., & Stadnick, N. A. (2023). Caregiver insights and improvement strategies for youth with autism undergoing Gastrointestinal endoscopy. *Journal of Autism and Developmental Disorders*, *53*(4), 1476–1482. 10.1007/s10803-021-05346-5 35217944 10.1007/s10803-021-05346-5 PMC9402801

[CR33] Kopecky, K., Broder-Fingert, S., Iannuzzi, D., & Connors, S. (2013). The needs of hospitalized patients with autism spectrum disorders: A parent survey. *Clin Pediatr (Phila)*, *52*(7), 652–660. 10.1177/0009922813485974 23624619 10.1177/0009922813485974

[CR34] Kouo, J. L., & Kouo, T. S. (2021). A scoping review of targeted interventions and training to facilitate medical encounters for School-Aged patients with an autism spectrum disorder. *Journal of Autism and Developmental Disorders*, *51*(8), 2829–2851. 10.1007/s10803-020-04716-9 33068218 10.1007/s10803-020-04716-9

[CR35] Lamptey, D. L. (2022). Navigating the Ghanaian health system: Stories from families of children with intellectual and developmental disabilities. *International Journal of Developmental Disabilities*, *68*(5), 641–650. 10.1080/20473869.2020.1865121 36210906 10.1080/20473869.2020.1865121 PMC9542259

[CR36] Leadbitter, K., Buckle, K. L., Ellis, C., & Dekker, M. (2021). Autistic Self-Advocacy and [perspective]he neurodiversity movement: Implications for autism [perspective]arly [perspective]ntervention [perspective]esearch and [perspective]ractice [Perspective]. *Frontiers in Psychology*, *12*. 10.3389/fpsyg.2021.635690 10.3389/fpsyg.2021.635690 PMC8075160 33912110

[CR37] Lord, C., Charman, T., Havdahl, A., Carbone, P., Anagnostou, E., Boyd, B., Carr, T., de Vries, P. J., Dissanayake, C., Divan, G., Freitag, C. M., Gotelli, M. M., Kasari, C., Knapp, M., Mundy, P., Plank, A., Scahill, L., Servili, C., Shattuck, P., & McCauley, J. B. (2022). The lancet commission on the future of care and clinical research in autism. *The Lancet*, *399*(10321), 271–334. 10.1016/S0140-6736(21)01541-5 10.1016/S0140-6736(21)01541-5 34883054

[CR38] Malik-Soni, N., Shaker, A., Luck, H., Mullin, A. E., Wiley, R. E., Lewis, M. E. S., Fuentes, J., & Frazier, T. W. (2022). Tackling healthcare access barriers for individuals with autism from diagnosis to adulthood. *Pediatric Research*, *91*(5), 1028–1035. 10.1038/s41390-021-01465-y 33767375 10.1038/s41390-021-01465-y PMC7993081

[CR40] Mason, D., Ingham, B., Urbanowicz, A., Michael, C., Birtles, H., Woodbury-Smith, M., Brown, T., James, I., Scarlett, C., Nicolaidis, C., & Parr, J. R. (2019). A systematic review of what barriers and facilitators prevent and enable physical healthcare services access for autistic adults. *Journal of Autism and Developmental Disorders*, *23*. 10.1007/s10803-019-04049-2 10.1007/s10803-019-04049-2 PMC6647496 31124030

[CR39] Mason, D., Ingham, B., Birtles, H., Michael, C., Scarlett, C., James, I. A., Brown, T., Woodbury-Smith, M., Wilson, C., Finch, T., & Parr, J. R. (2021). How to improve healthcare for autistic people: A qualitative study of the views of autistic people and clinicians. *Autism*, *25*(3), 774–785. 10.1177/1362361321993709 33910390 10.1177/1362361321993709

[CR41] Matusiak, M. (2016). *How to create an autism-friendly environment*. *Living Autism*. https://livingautism.com/create-autism-friendly-environment/

[CR42] Menendez, G. (2021). Supporting participation in healthcare: Patient and caregiver perspectives of an occupational therapy patient navigator (OTPN) for people with ASD/IDD. *Dissertation Abstracts International: Section B: The Sciences and Engineering*, *82*(4-B), No Pagination Specified. https://open.bu.edu/bitstream/handle/2144/41436/Menendez_bu_0017E_15972.pdf?sequence=7%26isAllowed=y

[CR43] Milton, D. E. M., Martin, N., & Melham, P. (2017). Beyond reasonable adjustment: autistic-friendly spaces and Universal Design. In *Autism and Intellectual Disability in Adults* (Vol. 1, pp. 81–85). Pavilion Publishing and Media Ltd. https://openresearch.lsbu.ac.uk/download/27cb2c4d1408ebe0465efa13f84357df9a7d8f4e4cdee2d54861a1dffbf0837a/74646/Autism%20Annual_E.pdf

[CR44] Morris, R., Greenblatt, A., & Saini, M. (2019). Healthcare providers’ experiences with autism: A scoping review. *Journal of Autism and Developmental Disorders*, *49*(6), 2374–2388. 10.1007/s10803-019-03912-6 30758692 10.1007/s10803-019-03912-6

[CR45] Munn, Z., Peters, M. D. J., Stern, C., Tufanaru, C., McArthur, A., & Aromataris, E. (2018). Systematic review or scoping review? Guidance for authors when choosing between a systematic or scoping review approach. *Bmc Medical Research Methodology*, *18*(1), 143. 10.1186/s12874-018-0611-x 30453902 10.1186/s12874-018-0611-x PMC6245623

[CR46] Muskat, B., Burnham Riosa, P., Nicholas, D. B., Roberts, W., Stoddart, K. P., & Zwaigenbaum, L. (2015). Autism comes to the hospital: The experiences of patients with autism spectrum disorder, their parents and health-care providers at two Canadian paediatric hospitals. *Autism*, *19*(4), 482–490. 10.1177/1362361314531341 24811967 10.1177/1362361314531341

[CR48] Nicholas, D. B., Zwaigenbaum, L., Muskat, B., Craig, W. R., Newton, A. S., Cohen-Silver, J., Sharon, R. F., Greenblatt, A., & Kilmer, C. (2016a). Toward Practice Advancement in Emergency Care for Children With Autism Spectrum Disorder. *Pediatrics*, *137 Suppl 2*, S205-211. 10.1542/peds.2015-2851S 10.1542/peds.2015-2851S 26908476

[CR49] Nicholas, D. B., Zwaigenbaum, L., Muskat, B., Craig, W. R., Newton, A. S., Kilmer, C., Greenblatt, A., Roberts, W., & Cohen-Silver, J. (2016b). Experiences of emergency department care from the perspective of families in which a child has autism spectrum disorder. *Social Work in Health Care*, *55*(6), 409–426. 10.1080/00981389.2016.1178679 27315287 10.1080/00981389.2016.1178679

[CR47] Nicholas, D. B., Muskat, B., Zwaigenbaum, L., Greenblatt, A., Ratnapalan, S., Kilmer, C., Craig, W., Roberts, W., Cohen-Silver, J., Newton, A., & Sharon, R. (2020). Patient- and Family-Centered care in the emergency department for children with autism. *Pediatrics*, *145*(Suppl 1), S93–s98. 10.1542/peds.2019-1895L 32238535 10.1542/peds.2019-1895L

[CR50] O’Hagan, B., Krauss, S. B., Friedman, A. J., Bartolotti, L., Abubakare, O., Broder-Fingert, S., & Augustyn, M. (2023). Identifying components of autism friendly health care: An exploratory study using a modified Delphi method. *Journal of Developmental & Behavioral Pediatrics*, *44*(1), e12–e18. 10.1097/dbp.0000000000001139 36367772 10.1097/DBP.0000000000001139

[CR51] Peters, M. D. J., Marnie, C., Tricco, A. C., Pollock, D., Munn, Z., Alexander, L., McInerney, P., Godfrey, C. M., & Khalil, H. (2021). Updated methodological guidance for the conduct of scoping reviews. *JBI Evidence Implementation*, *19*(1), 3–10. 10.1097/xeb.0000000000000277 33570328 10.1097/XEB.0000000000000277

[CR52] Pillai, J., Dunn, K., & Efron, D. (2023). Parent-reported factors associated with the emergency department presentation of children and adolescents with autism spectrum disorder and/or intellectual disability with behaviours of concern: A qualitative study. *Archives of Disease in Childhood*, *108*(4), 264–270. 10.1136/archdischild-2022-325002 36521861 10.1136/archdischild-2022-325002

[CR53] Roche, L., Adams, D., & Clark, M. (2021). Research priorities of the autism community: A systematic review of key stakeholder perspectives. *Autism*, *25*(2), 336–348. 10.1177/1362361320967790 33143455 10.1177/1362361320967790

[CR54] Rotella, J. A. (2022). No one brain is the same: A neurodivergent clinician’s approach to caring for the neurodivergent patient in the emergency department. *Emergency Medicine Australasia*, *34*(4), 613–615. 10.1111/1742-6723.14039 35764294 10.1111/1742-6723.14039

[CR55] Saeed, G., Brown, H. K., Lunsky, Y., Welsh, K., Proulx, L., Havercamp, S., & Tarasoff, L. A. (2022). Barriers to and facilitators of effective communication in perinatal care: A qualitative study of the experiences of birthing people with sensory, intellectual, and/or developmental disabilities. *Bmc Pregnancy and Childbirth*, *22*(1), 364. 10.1186/s12884-022-04691-2 35473673 10.1186/s12884-022-04691-2 PMC9044670

[CR56] Samet, D., & Luterman, S. (2019). See-Hear-Feel-Speak: A protocol for improving outcomes in emergency department interactions with patients with autism spectrum disorder. *Pediatric Emergency Care*, *35*(2), 157–159. 10.1097/pec.0000000000001734 30702545 10.1097/PEC.0000000000001734

[CR57] Samuel, P., Yew, R. Y., Hooley, M., Hickey, M., & Stokes, M. A. (2022). Sensory challenges experienced by autistic women during pregnancy and childbirth: A systematic review. *Archives of Gynecology and Obstetrics*, *305*(2), 299–311. 10.1007/s00404-021-06109-4 34085111 10.1007/s00404-021-06109-4

[CR58] Shady, K., Phillips, S., & Newman, S. (2022). Barriers And facilitators to healthcare access in adults with intellectual And developmental disorders And communication difficulties: An integrative review. *Rev J Autism Dev Disord*, 1–13. 10.1007/s40489-022-00324-8 10.1007/s40489-022-00324-8 PMC9148936 35669718

[CR59] Snow, S. L., Smith, I. M., Latimer, M., Stirling Cameron, E., Fox, J., & Chorney, J. (2022). A balancing act: An interpretive description of healthcare providers’ and families’ perspective on the surgical experiences of children with autism spectrum disorder. *Autism*, *26*(4), 839–848. 10.1177/13623613211034057 34320870 10.1177/13623613211034057 PMC9014760

[CR60] Straus, J. A. O., Coburn, S., Maskell, S., Pappagianopoulos, J., & Cantrell, K. (2019). Medical encounters for youth with autism spectrum disorder: A comprehensive review of environmental considerations and interventions. *Clinical Medicine Insights Pediatrics*, (13), 1179–5565. 10.1177/1179556519842816 10.1177/1179556519842816 PMC6488780 31065222

[CR61] Taghizadeh, N., Heard, G., Davidson, A., Williams, K., & Story, D. (2019). The experiences of children with autism spectrum disorder, their caregivers and health care providers during day procedure: A mixed methods study. *Paediatric Anaesthesia*, *29*(9), 927–937. 10.1111/pan.13689 31448870 10.1111/pan.13689

[CR62] Tricco, A. C., Lillie, E., Zarin, W., O’Brien, K. K., Colquhoun, H., Levac, D., Moher, D., Peters, M. D. J., Horsley, T., Weeks, L., Hempel, S., Akl, E. A., Chang, C., McGowan, J., Stewart, L., Hartling, L., Aldcroft, A., Wilson, M. G., Garritty, C., & Straus, S. E. (2018). PRISMA extension for scoping reviews (PRISMA-ScR): Checklist and explanation. *Annals of Internal Medicine*, *169*(7), 467–473. 10.7326/m18-0850 30178033 10.7326/M18-0850

[CR63] Victorian Health Building Authority (2022, 23 February). *Universal design*. Victorian Health Building Authority. Retrieved September 22 2024 from https://www.vhba.vic.gov.au/resources/universal-design

[CR64] Vohra, R., Madhavan, S., & Sambamoorthi, U. (2016). Emergency department use among adults with autism spectrum disorders (ASD). * Journal of autism and developmental disorder*, 46(4), 1441–1454. 10.1007/s10803-015-2692-2 10.1007/s10803-015-2692-2 PMC4845033 26762115

[CR65] Walsh, C., Lydon, S., Hehir, A., & O’Connor, P. (2020a). Development and evaluation of a novel caregiver-report tool to assess barriers to physical healthcare for people on the autism spectrum. *Res Autism Spectr Disord*, *79*, 101680. 10.1016/j.rasd.2020.101680 33072182 10.1016/j.rasd.2020.101680 PMC7554131

[CR66] Walsh, C., Lydon, S., O’Dowd, E., & O’Connor, P. (2020b). Barriers to healthcare for persons with autism: A systematic review of the literature and development of A taxonomy. *Dev Neurorehabil*, *23*(7), 413–430. 10.1080/17518423.2020.1716868 36112897 10.1080/17518423.2020.1716868

[CR67] Walsh, C., Connor, O., Walsh, P., E., & Lydon, S. (2021). A systematic review of interventions to improve healthcare experiences and access in autism. *Review Journal of Autism and Developmental Disorders*, *10*, 1–18. 10.1007/s40489-021-00279-2

[CR68] Westaway, A. (2023). Parents’ experiences of how healthcare professionals communicate with autistic children. *Child Care in Practice*, *29*(1), 68–82. 10.1080/13575279.2022.2119939

[CR69] Wilson, S. A., & Peterson, C. C. (2018). Medical care experiences of children with autism and their parents: A scoping review. *Child: Care Health and Development*, *44*(6), 807–817. 10.1111/cch.12611 30136407 10.1111/cch.12611

[CR70] Wood, E. B., Halverson, A., Harrison, G., & Rosenkranz, A. (2019). Creating a Sensory-Friendly pediatric emergency department. *Journal of Emergency Nursing*, *45*(4), 415–424. 10.1016/j.jen.2018.12.002 30679010 10.1016/j.jen.2018.12.002

